# Interest of the Addition of Taxanes to Standard Treatment in First-Line Advanced HER2 Positive Gastroesophageal Adenocarcinoma in Selective Patients

**DOI:** 10.3389/fonc.2022.763926

**Published:** 2022-03-07

**Authors:** Emeline Orillard, Julie Henriques, Dewi Vernerey, Hamadi Almotlak, Fabien Calcagno, Francine Fein, Serge Fratté, Marine Jary, Elodie Klajer, Angelique Vienot, Christophe Borg, Stefano Kim

**Affiliations:** ^1^ Department of Oncology, University Hospital of Besançon, Besançon, France; ^2^ Bourgogne Franche-Comté University, INSERM, Établissement Français du Sang Bourgogne Franche-Comté, UMR1098, Interactions Hôte-Greffon-Tumeur/Ingénierie Cellulaire et Génique, Besançon, France; ^3^ Methodology and Quality of Life Unit in Oncology, University Hospital of Besançon, Besançon, France; ^4^ Department of Gastroenterology, University Hospital of Besançon, Besançon, France; ^5^ Department of Gastroenterology, Centre Hospitalier Régional, Belfort, France; ^6^ Department of Oncology and Radiotherapy, Hôpital Nord-Franche Comté, Montbéliard, France

**Keywords:** taxanes, HER2, gastric carcinoma, metastatic, trastuzumab, survival

## Abstract

**Background:**

Studies have reported a beneficial role of the addition of trastuzumab to platin-5-FU based chemotherapy in first-line advanced HER2 positive gastroesophageal adenocarcinoma (GEA). However, the effect of taxanes combined with platin-5FU + trastuzumab (PFT) is understudied.

**Methods:**

We performed a retrospective cohort study to evaluate the interest of taxanes among HER2-positive advanced GEA patients treated with PFT. We enrolled HER2-positive advanced GEA patients who underwent treatment between January 2009 to March 2021 in seven hospitals centers in France, treated with PFT alone (S group) or with taxanes + PFT regimen (T group). The primary outcome was progression-free survival (PFS). Also, overall survival (OS), response rate, conversion surgery rate, and safety were evaluated.

**Results:**

Overall, 65 patients received PFT-based therapy, 24 patients in the T group, and 41 patients in the S group. To avoid the selection bias, only those patients presenting an ECOG-PS of 0-1 and synchronous metastasis (21 patients in the T group and 19 patients in the S group) were included for analysis. The median PFS was 9.3 months (95%CI 7.0 to 17.2) in the T group and 5.9 months (95%CI 3.7 to 9.6) in the S group (log-rank p=0.038). Treatment by taxanes was significantly associated with a better PFS in univariate (HR 0.49; 95%CI 0.25 to 0.98, p=0.042) and multivariate Cox regression analysis (HR 0.44; 95%CI 0.21 to 0.94, p=0.033), and IPTW method (HR 0.56; 95% CI 0.34 to 0.91, p=0.019). OS was prolonged (19.0 months (95%CI 7.8 to 45.2) vs 13.0 months (95%CI 5.5 to 14.8), log-rank p=0.033) in favor of the T group. Treatment by taxanes was significantly associated with a better OS in univariate Cox regression analysis (HR 0.49; 95%CI 0.21 to 0.96, p=0.038) and IPTW method (HR 0.49; 95% CI 0.29 to 0.84, p=0.009). The response rate was higher in the T group, with conversion surgery in five patients. No treatment-related death was observed in both groups.

**Conclusions:**

Given the improvement in PFS and OS, the addition of taxanes to standard chemotherapy could be considered as a promising treatment for selected HER2-positive advanced GEA patients, with PS 0-1 and synchronous metastasis (NCT04920747).

## Introduction

Fluoropyrimidine and platinum-based chemotherapy, in association with trastuzumab, an anti-human epidermal growth factor receptor 2 (HER2) monoclonal antibody, is the standard of care in HER2-positive advanced gastroesophageal adenocarcinoma (GEA) patients, based on the results of the ToGA study published in 2010 (1). Then, all additional efforts to improve anti-HER2 inhibition in first- and second-line, failed to prolong survival. In the second line, trastuzumab maintenance beyond progression, in combination with paclitaxel, failed to improve PFS compared to paclitaxel alone (2). Besides, lapatinib (a tyrosine kinase inhibitor of epidermal growth factor receptor HER1 and HER2) plus paclitaxel (3), or trastuzumab-emtansine (TDM-1), an antibody-drug conjugate (4), also failed to improve survival compared to paclitaxel alone in phase III trials. In first-line, the JACOB trial group has proposed to intensity anti-HER2 blockade with the addition of pertuzumab to trastuzumab and standard chemotherapy (5), and the HELOISE trial group proposed to increase the dose of trastuzumab (6). Unfortunately, both phase III trials were negative, and no survival improvement was seen in HER2-positive GEA patients for 10 years. Recently, in an open-label randomized phase II trial, a new antibody-drug conjugate trastuzumab-deruxtecan showed encouraging results in third- or later line with a significant improvement in objective response rate (51% vs 14%; p<0.001) and overall survival (HR, 0.59; p=0.01) (7). A confirmatory phase III trial is ongoing (DESTINY-Gastric04).

These negative results are contrary to what we have seen in HER2-positive breast cancer, for which the intensification of HER2 inhibition in first-line, as well as, the continuous exposition to an anti-HER2 therapy remains a standard of care in metastatic HER2-positive breast cancer patients setting. Several issues had been highlighted in GEA as possible explanations, like the tumor heterogeneity (8) or the presence of molecular mechanisms of resistance to anti-HER2 therapy, such as changes in the tridimensional receptor structures, the co-expression of other transmembrane receptors, or the activation of downstream signaling effectors (9). Moreover, in the case of secondary resistance to anti-HER2 inhibitor, the loss of HER2 expression has been described (2, 10). In fact, among those patients with new tumor biopsy at progression under trastuzumab in first-line, about 60-70% of patients have lost the HER2 expression in their tumors.

**Figure 1:**
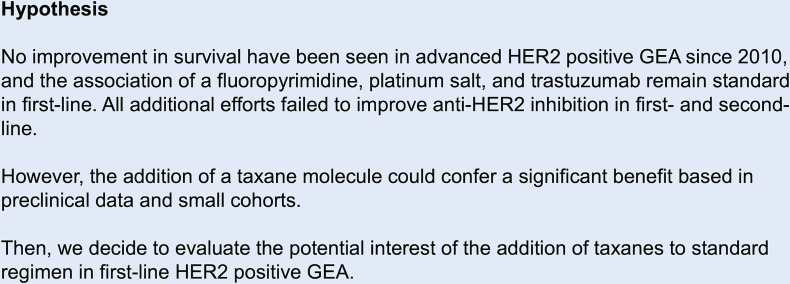
The hypothesis to combine taxanes to standard treatment in first-line advanced HER2 positive gastroesophageal adenocarcinoma.

To date, the prognosis of HER2-positive GEA patients remains bleak, with an estimated median overall survival of 14 months after the introduction of the first-line therapy (1, 5). Therefore, there is still an unmet need in this high-risk population. Considering the lack of efficacy of anti-HER2-based intensification therapy, it seems reasonable to optimize the first-line treatment of these patients with a synergic chemotherapy. Our hypothesis is presented in **Figure 1**. The rationale to combine taxanes to the standard regimen in HER2-positive advanced GEA is based on I) their demonstrated efficacy in the first line in HER2-negative GEA patients, and II) the synergic effect in preclinical and clinical settings between trastuzumab and taxanes-based chemotherapy in HER2-positive tumors. In a phase III trial, Van Cutsem et al. (11) have shown in these patients that the addition of docetaxel to cisplatin and 5-Fluorouracil (standard DCF) as the first-line therapy significantly improved time to progression, overall survival, and response rate. More recently, a randomized phase II study has compared dose-modified docetaxel, cisplatin, and 5-FU (mDCF) to standard DCF regimen in previously untreated patients with advanced GEA (12). Toxicity was significantly decreased with mDCF with improved efficacy and was recommended as a new option for first-line therapy (13). In the preclinical model of breast cancer cell lines, the efficacy of trastuzumab in combination with different types of chemotherapy was evaluated. It was observed synergistic interaction between the use of trastuzumab and docetaxel, and an additive interaction between trastuzumab and paclitaxel (14–16), including an enhancement of trastuzumab-mediated antibody-dependent cellular cytotoxicity (ADCC) on tumor cells by taxanes through NKG2D-mediated NK cell recognition (17). In HER2-positive GEA patients in first-line, Al Batran et al. have shown for the first time in 2012 a report of 3 cases of patients that adding trastuzumab to a docetaxel-based, triple-drug chemotherapy combination is feasible and highly active (18). Since then, several single-arm small phase II trials evaluated the association of taxanes, platin salt, 5-FU, and trastuzumab regimens (19–21). In these studies, including 15 to 26 patients, this combination was feasible, and the mPFS was 9 to 13 months. One study had mPFS not reached during the median follow-up time of 18.3 months. The overall response rate was 60% to 93%.

Thus, to evaluate the potential interest of the addition of taxanes to trastuzumab and standard chemotherapy for HER2-positive advanced GEA in the first line, we performed a retrospective multicenter TanDHER study to compare taxanes, platin, 5-FU, and trastuzumab (TPFT) regimen with standard chemotherapy and trastuzumab.

## Patients and Methods

### Patient Selection

TanDHER (NCT04920747) was a retrospective study undertaken in 7 centers in the Franche-Comte region in France. Patients treated between January 2009 and Mars 2021 were included. Men or women older than 17 years of age were eligible for inclusion if they had histologically confirmed (by tumor biopsy or on a surgical specimen and confirmed by the pathologists of each center) non-resectable locally advanced, recurrent, or metastatic adenocarcinoma of the stomach or gastro-esophageal junction; tumor samples scored as 3+ on immunohistochemistry (IHC) or a FISH (Fluorescence *in situ* Hybridization) positive tumor (HER2:CEP17 ratio ≥2) with a 2+ IHC score, based on This criterion is based on the guideline from the College of American Pathologists, American Society for Clinical Pathology, and American Society of Clinical Oncology regarding HER2 assessment in gastroesophageal adenocarcinoma (22, 23); measurable disease; treated by trastuzumab, platin (cisplatin or oxaliplatin), fluoropyrimidine (5-FU or capecitabine) +/- taxanes (docetaxel or paclitaxel), as first-line therapy for advanced gastric cancer. Major exclusion criteria included previous chemotherapy by taxane for metastatic disease and previous anti-HER2 therapy. The study was done in accordance with Good Clinical Practice guidelines and the Declaration of Helsinki. All patients provided written informed consent. Approvals for the study protocol were obtained from the independent ethic committee (Clinical Ethics Committee, CHRU Besancon, 03/04/2019).

### Treatment

Treatments were classified as TPFT (taxanes-platin-fluoropyrimidine-trastuzumab) regimen if patients received at least one cycle of this protocol, and standard (S) regimen if they received at least one cycle of platin-fluoropyrimidine-trastuzumab treatment with the exclusion of taxanes. TPFT regimen consisted of docetaxel (75 mg/m2 on day 1), cisplatin (75 mg/m2 on day 1), and 5-fluorouracil (750 mg/m2/day on continuous perfusion on days 1-5), every 3 weeks as previously described by Van Cutsem et al. (standard DCF regimen) or docetaxel (40 mg/m2 IV on day 1), cisplatin (40 mg/m2 IV on day 3) 5-fluorouracil (2000 mg/m2 intravenously over 48 hours) every 2 weeks as previously described by Shah et al. (modified DCF regimen).

Trastuzumab was given by intravenous infusion at a dose of 8mg/kg on day 1 of the first cycle, followed by 6 mg/kg every 3 weeks in association with standard DCF; or 6mg/kg on day 1 of the first cycle, followed by 4 mg/kg every 2 weeks in association with modified DCF. S regimen corresponds to the regimen defined in the ToGA trial or the combination of oxaliplatin and fluoropyrimidine (5-FU or capecitabine).

### Conversion Therapy

In case of disease control rate (complete or partial response, or stable disease), a multidisciplinary team comprising medical oncologists and surgeons re-evaluated all patients to determine potential conversion therapy. This strategy was defined as a local treatment aiming at an R0 resection after chemotherapy in initially unresectable stage IV gastric cancer. In this study, it was composed of the resection of the primary tumor and local treatment (surgery, radiotherapy, cryotherapy, or radiofrequency) of metastasis.

### Definition of Variables

The primary outcome was progression-free survival (PFS), defined as the time interval between the start of the treatment and the clinical or radiological progression, or death from any cause. Alive patients free of progression were censored at the last date of news. Clinical records were used to obtain baseline characteristics: gender, age at diagnosis, tumor localization, TNM classification (by the 2009 classification), characteristics of localized surgical management, (neo)adjuvant chemotherapy or radiotherapy, perioperative treatment, metastatic recurrence, and management.

Secondary outcomes were overall survival (OS), defined as the time interval between the start of the treatment and the death from any cause; the objective response rate (ORR) and the disease control rate (DCR) evaluated using Response Evaluation Criteria in Solid Tumors (RECIST) criteria version 1.1; the secondary resectability of primary tumor or metastasis in patients with DCR (complete or partial response, or stable disease); and the safety.

### Statistical Analysis

Qualitative variables were described using frequency and percentage, and continuous variables were described using median and range. These characteristics were compared statistically using Chi2 or Fisher tests for categorical variables. Quantitative variables were compared statistically using a Wilcoxon test. Median follow-up with a 95% confidence interval (CI) was estimated with the reverse Kaplan Meier method. PFS and OS were assessed using a Kaplan Meier method, described with median and 95% confidence interval (CI), and the survivals between the two groups were compared using a log-rank test. Association between baseline characteristics and PFS and OS was investigated with Cox proportional hazards regression model and hazard ratio (HR) with 95%CI were provided. Variables with p-value < 0.15 in univariate analysis were entered in a multivariate model, and the treatment arm was forced. In order to limit bias due to potential confounding factors unbalanced between treatment groups, the inverse of probability of treatment weighting (IPTW) (24) method was applied in a univariate Cox model using the propensity score. This propensity score was derived from a multivariate logistic regression which estimates the probability to be in the T group using variables with p-value <0.15 in univariate logistic regression. Analysis of survival in the subgroup of patients’ ECOG PS 0-1 and synchronous metastasis was performed. We have also compared the secondary resectability between the two groups. Statistical analyses were performed under SAS version 9.4 considering a significance threshold α of 5%. These statistical analyses were carried out by the Methodology and Quality of Life in Cancerology Unit of the Besançon University Hospital (UMR 1098), based on data collected within the e-CRF.

## Results

### Patients’ Characteristics in the Overall Population

Clinical characteristics of the 65 evaluable patients are listed in **Supplementary Table 1**. Twenty-four patients (37%) received TPFT regimen (T group), and 41 patients (63%) received standard regimen (S group), with no taxanes. The median age was 68.7 (range, 30.3-90.6) and 75.4% had a synchronous metastatic disease at diagnosis. Sixty-three percent of patients had gastric tumors, and approximately 75% of the tumors have moderately or poorly differentiated histology. The population was relatively high functioning in both groups (ECOG performance status (PS) 0 or 1: 86% of patients). Nevertheless, 20% of patients presented PS of 2 on the ECOG scale in the S group, compared to 4.2% in the T group (p=0.14). Patients’ characteristics were similar in both groups, except for age [64.5 (39-79.3) in the T group versus 72.8 (30.3-90.6) for the S group (p=0.015)] and metastatic disease with no surgery of primary tumor (91.7% in T group vs 65.9% in S group, p=0.02) at diagnosis (**Supplementary Table 1**).

### Selection of the Population of Analysis: Patients With ECOG PS 0-1 and Synchronous Metastasis

**Figure 2:**
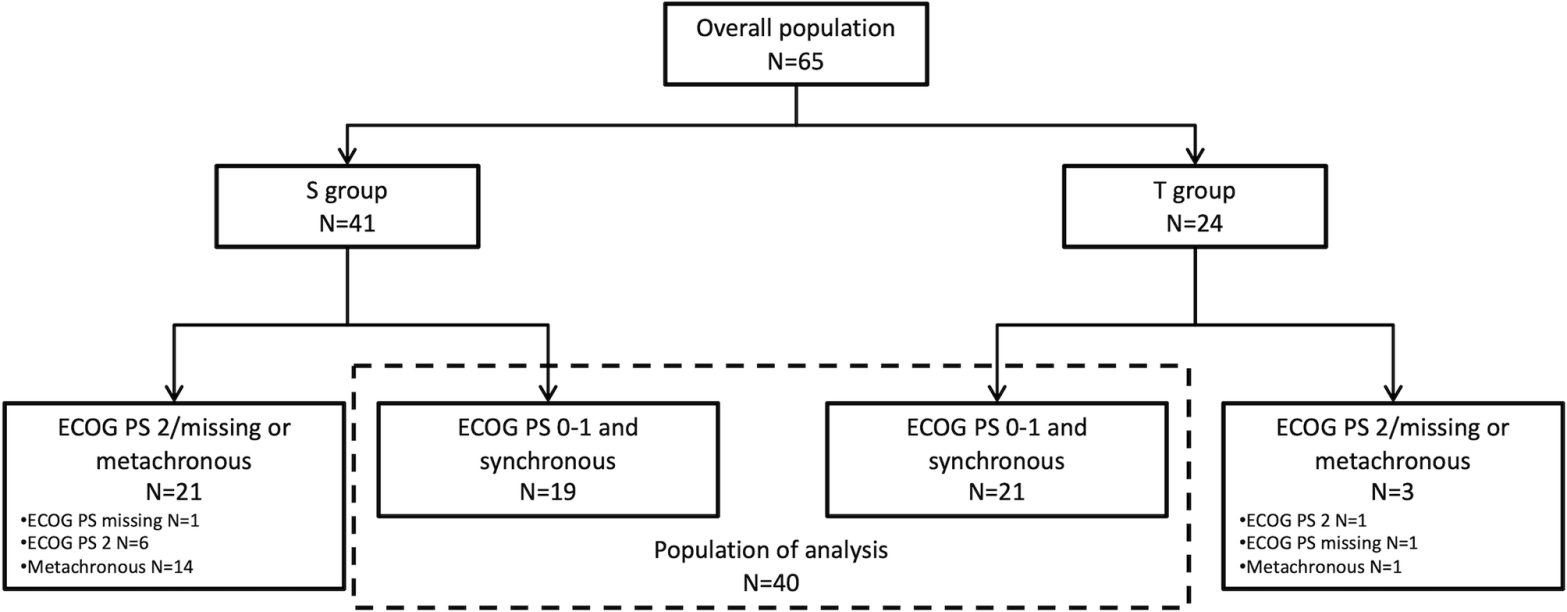
Flowchart. S group: Standard regimen. T group: TPFT regimen. PS, Performance status.

To study groups of patients with similar clinical characteristics, we decided to select patients with ECOG PS 0-1 and synchronous metastasis (**Figure 2**). Baseline characteristics of patients in the population of analysis were presented in **Table 1**; composed of 19 patients in the S group and 21 patients in the T group. The median age was 71.2 (range, 30.3-90.6) in the S group and 64.2 (range, 39-79.3) in the T group (p=0.035). Other patients’ characteristics were similar in both groups. In the case of taxanes based regimen, 16 patients received the mDCF regimen, 4 patients received the DCF regimen, and 1 patient received the FLOT regimen (**Table 1**).

**Table 1:** Baseline characteristics of patients in the population of analysis.

		Total (n = 40)		S group (n = 19)		T group (n = 21)		p-value
		n	%	n	%	n	%	
**Age**	Median	67.55		71.23		64.23		**0.0346**
	Range	30.34-90.56		30.34-90.56		39.00-79.3		
**Gender**	Male	34	85	16	84.21	18	85.71	1
	Female	6	15	3	15.79	3	14.29	
**ECOG PS**	0	21	52.5	9	47.37	12	57.14	0.5365
	1	19	47.5	10	52.63	9	42.86	
**Initial Diagnosis**								
**Primary tumor**	Stomach	27	67.5	15	78.95	12	57.14	0.1415
	GEJ/Lower esophagus	13	32.5	4	21.05	9	42.86	
**Metastasis location**								
**Peritoneum**	No	31	77.5	16	84.21	15	71.43	0.457
	Yes	9	22.5	3	15.79	6	28.57	
**Bones**	No	35	87.5	17	89.47	18	85.71	1
	Yes	5	12.5	2	10.53	3	14.29	
**Lung**	No	32	80	14	73.68	18	85.71	0.442
	Yes	8	20	5	26.32	3	14.29	
**Brain**	No	38	95	17	89.47	21	100	0.2192
	Yes	2	5	2	10.53	0	0	
**Liver**	No	13	32.5	6	31.58	7	33.33	0.9058
	Yes	27	67.5	13	68.42	14	66.67	
**Other sites**	No	38	95	19	100	19	90.48	0.4885
	Yes	2	5	0	0	2	9.52	
**Pathology Characteristics**								
**Histology**	Well differentiated	10	28.57	6	35.29	4	22.22	0.7548
	Moderately/Un-differentiated	25	71.43	11	64.71	14	77.78	
	Missing	5	-	2	-	3	-	
**HER2 Status**	HER2 3+	34	85.0	15	78.95	19	90.48	0.3976
	HER2 2+/FISH+	6	15.0	4	21.05	2	9.52	
**First line chemotherapy regimes**								
	Modified DCF	–	–	–	–	16	76	
	Standard DCF	–	–	–	–	4	19	
	FLOT	–	–	–	–	1	5	
	FOLFOX	–	–	14	74	–	–	
	CDDP-5FU	–	–	3	16	–	–	
	TOMOX	–	–	2	10	–	–	
**Second line systemic cancer therapy**								
**Patients**		23	57.5	12	63.16	11	52.38	0.4911
**Types of treatment**	Paclitaxel	–	–	9	75	2	18.2	
	FOLFIRI	–	–	1	8.3	5	45.4	
	DCFm	–	–	–		1	9.1	
	FOLFOX	–	–	2	16.7	1	9.1	
	EOX	–	–	–		1	9.1	
	MAC	–	–	–		1	9.1	

Population of analysis: patients with ECOG PS 0 - 1 and synchronous metastasis. S group: Standard regimen. T group: TPFT regimen. DCF, Docetaxel Cisplatin 5-Fluorouracil. EOX, Epirubicin; Oxaliplatin; and Capecitabine; FISH, Fluorescence in situ Hybridization; 5-FU, 5-Fluorouracil; FLOT, 5-Fluorouracil, Folinic acid, oxaliplatin and docetaxel. FOLFIRI, Folic acid, 5-Fluorouracil and Irinotecan; FOLFOX, Folinic acid, 5-Fluorouracil and Oxaliplatin. GEJ, gastroesophageal junction; HER2, Human Epidermal Growth Factor Receptor 2. MAC, methotrexate, doxorubicin, and cisplatin; PS, Performans status; TOMOX, Raltitrexed and Oxaliplatin. Bold text: significant p-value.

### Outcomes in the Population of Analysis

**Figure 3:**
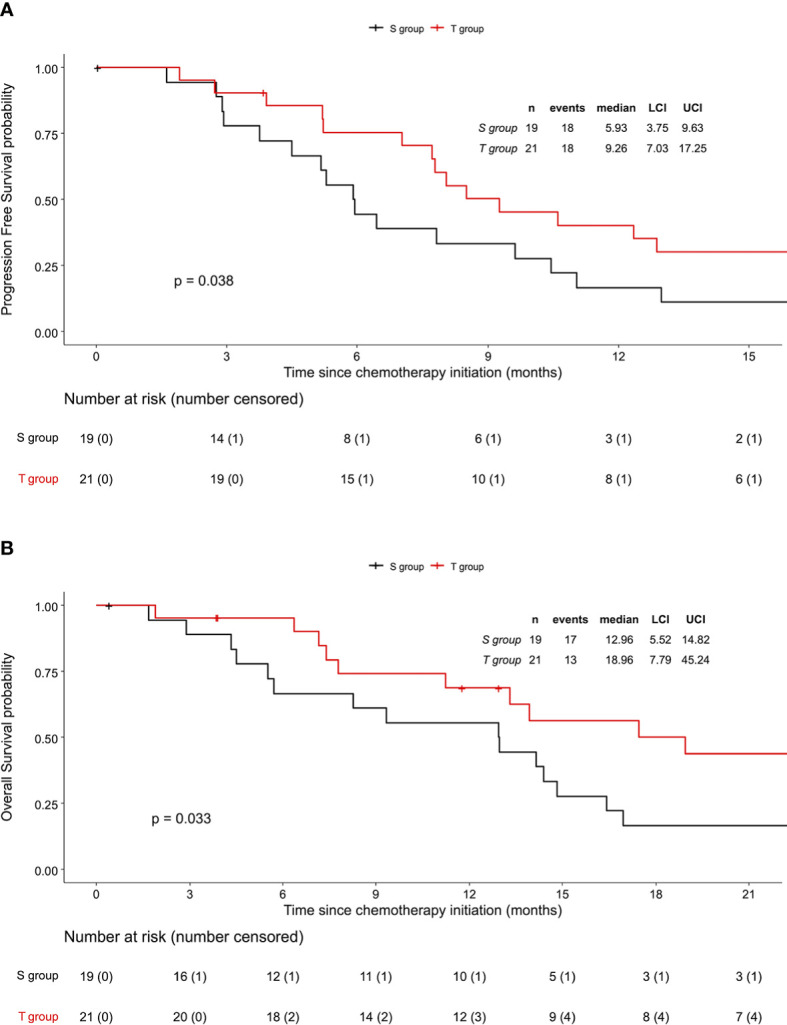
Outcomes according to the treatment group in the population of analysis. Population of analysis: patients with ECOG PS 0 - 1 and synchronous metastasis. **(A)** Progression-Free Survival and **(B)** Overall Survival. S group: Standard regimen. T group: TPFT regimen. CI, Confidence Interval.

In patients with ECOG PS 0-1 and synchronous metastasis (the population of analysis), the median PFS was 9.3 months (95%CI 7.0 to 17.2) in the T group and 5.9 months (95%CI 3.7 to 9.6) in the S group (log-rank p=0.038) (**Figure 3A**). Results from univariate and multivariate Cox regression analysis are presented in **Table 2**. In this analysis, no clinical or pathological characteristics were associated with PFS. Treatment by taxanes was significantly associated with a better PFS in univariate (HR 0.49; 95%CI 0.25 to 0.98, p=0.042) and multivariate analysis (HR 0.44; 95%CI 0.21 to 0.94, p=0.033).

**Table 2:** Univariate and multivariate Cox regression for PFS in the population of analysis.

		Univariate Cox regression				Multivariate Cox regression			
		n (events)	HR	95%CI	p-value	n (events)	HR	95%CI	p-value
**Gender**	**Male**	34 (30)	1		0.9242				
	**Female**	6 (6)	1.04	0.43-2.54					
**Age at treatment initiation**	**Continuous**	40 (36)	1.02	1.00-1.05	0.103				
	**<70**	22 (20)	1		0.1274	22 (20)	1		0.6909
	≥**70**	18 (16)	1.69	0.87-3.31		18 (16)	1.16	0.55-2.46	
**ECOG PS**	**0**	21 (19)	1		0.142	21 (19)	1		0.1001
	**1**	19 (17)	1.64	0.85-3.17		19 (17)	1.86	0.89-3.91	
**Initial Diagnosis**									
**Primary tumor**	**Stomach**	27 (26)	1		0.8953				
	**GEJ/lower esophagus**	13 (10)	0.95	0.46-1.98					
**Histology**	**Well**	10 (8)	1		0.2965				
	**Moderately/undifferentiated**	25 (25)	1.54	0.69-3.44					
**Metastasis location**									
**Peritoneum**	**No**	31 (28)	1		0.2394				
	**Yes**	9 (8)	1.61	0.73-3.57					
**Lymph nodes**	**No**	17 (16)	1		0.8914				
	**Yes**	23 (20)	0.96	0.49-1.85					
**Lung**	**No**	32 (29)	1		0.4712				
	**Yes**	8 (7)	0.73	0.31-1.71					
**Liver**	**No**	13 (11)	1		0.2355				
	**Yes**	27 (25)	1.55	0.75-3.18					
**Taxanes**	**No**	19 (18)	1		**0.0418**	19 (18)	1		**0.033**
	**Yes**	21 (18)	0.49	0.25-0.98		21 (18)	0.44	0.21-0.94	

Population of analysis: patients with ECOG PS 0 - 1 and synchronous metastasis. CI, Confidence Interval; HR, Hazard Ratio; PS, Performance Status. Bold text: significant p-value.

The median OS was 19.0 months (95%CI 7.8 to 45.2) in the T group and 13.0 months (95%CI 5.5 to 14.8) in the S group (log-rank p=0.033) (**Figure 3B**). In univariate Cox regression analysis, no baseline characteristics were associated with a better OS (**Supplementary Table 2**). Treatment by taxanes was significantly associated with a better OS (HR 0.45; 95%CI 0.21 to 0.96, p=0.0378) in univariate analysis, but not in multivariate analysis (HR 0.47; 95%CI 0.21 to 1.06, p=0.07).

### Propensity Score

A propensity score, derived from multivariate logistic regression analysis was performed to estimate the probability to have taxanes-based chemotherapy in patients with synchronous metastasis and ECOG PS 0-1. Age and tumor localization were added in the multivariate logistic regression (**Supplementary Table 3**).

According to the IPTW method applied in univariate Cox analysis, the use of taxanes was associated with better PFS (HR 0.56; 95%CI 0.34 to 0.91, p=0.0187) and OS (HR 0,49; 95%CI 0.29 to 0.84, p=0.009) in the population of analysis (**Table 3**).

**Table 3:** IPTW methodology applied in univariate Cox analysis for PFS and OS in the population of analysis.

			n (events)	HR	95%CI	p-value
**PFS**	**Taxanes**	**No**	19 (18)	1		**0.0187**
		**Yes**	21 (18)	0.56	0.34-0.91	
**OS**	**Taxanes**	**No**	19 (17)	1		**0.009**
		**Yes**	21 (13)	0.49	0.29-0.84	

Population of analysis (patients with ECOG PS 0 - 1 and synchronous metastasis). CI, Confidence Interval; HR, Hazard Ratio; PFS, Progression-free Survival; OS, Overall Survival. Bold text: significant p-value.

### Objective Response Rate and Conversion Therapy in the Population of Analysis

**Figure 4:**
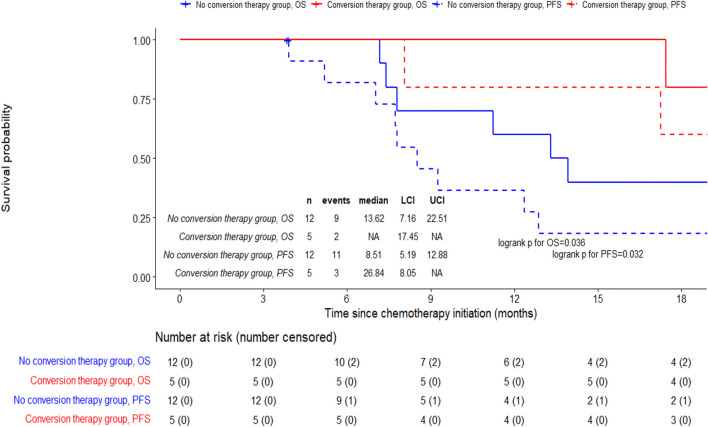
Outcomes according to conversion therapy in the T group of the population of analysis. Evaluation of progression-free survival and overall survival in the T group of the population of analysis (patients with ECOG PS 0 - 1 and synchronous metastasis). Conversion therapy corresponds to the resection of the primary tumor. Patients with conversion therapy have disease control after chemotherapy and local treatment of metastasis. CI, Confidence Interval; PFS, Progression-Free Survival; OS, Overall Survival.

In patients with ECOG PS 0-1 and synchronous metastasis (the population of analysis), the objective response rate was 68.4% in the T group versus 47.4% in the S group (p=0.19) (**Supplementary Table 4**). In the T group, 5 patients had surgical resection of the primary tumor (29.4%) compared to 1 patient in the S group (p=0.35) (**Supplementary Table 5**). Median PFS was 26.8 months (95%CI 8.0 to not reached (NR)) in case of surgical resection of the primary tumor, and 8.5 months (95%CI 5.2 to 12.9) in patients without surgical resection (log-rank 0.032). Median OS was not reached (95%CI 17.4 to NR) in case of surgical resection of the primary tumor, versus 13.6 months (95%CI 7.2 to 22.5) in patients without surgical resection (log-rank 0.0992) (**Figure 4**). Characteristics of patients who underwent conversion surgery were described in **Supplementary Table 6**.

### Patients’ Exposure to Treatment and Toxicity in the Population of Analysis

In patients with ECOG PS 0-1 and synchronous metastasis, the median number of cycles was 8 (range, 2–16) in the T group compared to 9 in the S group (range, 1-15) (p=0.9566). The rate of patients with a dose reduction of one of the chemotherapy molecules during the entire course of treatment was 62% in the T group versus 74% in the S group (p=0.427). There was no difference in early dose reduction (cycle 1 to 3) between both groups (29% in the T group and 37% in the S group, p=0.577). No toxic death was observed in both groups (**Supplementary Table 7**).

## Discussion

After the ToGA trial, the combination of platin-5-FU chemotherapy and trastuzumab became the standard of care in HER2-positive advanced GEA patients. In this study, we found that the combination of the addition of docetaxel to platin, 5-FU, and trastuzumab (TPFT) have a potential interest in selected patients. A survival advantage was observed in favor of the TPFT regimen, and this finding was consistent across different analytic approaches.

Despite the small number of patients, median PFS and OS in the S group remain comparable to the ToGA study, with 5.6 and 12.9 months, respectively in our study compared to 6.7 and 13.8 months in the ToGA study (1). In the S group, the ORR seems to be also similar, evaluated to 37% in our study and 47% in the ToGA study. In our T group, median PFS and OS were 9.3 and 19.0 months, respectively. Then, these results are comparable to single-arm small phase II trials evaluating taxanes, platin, 5-FU, and trastuzumab regimens (**Table 4**). The mPFS was 9.2 months to not reached, and mOS was 19.4 to not reached with a median follow up to 18.3 months (19–21). More recently, a retrospective study including 22 patients treated with trastuzumab plus taxane-based chemotherapy, and 108 patients treated with trastuzumab plus standard chemotherapy as first-line treatment in HER2-positive advanced GEA patients, showed a significant improvement in OS in favor of taxanes. The mOS was 15.2 months (95% CI 12.7-17.7) versus 11.1 months (95% CI 8.3-13.9; p = 0.03) (25), in line with our results.

**Table 4:** Main outcomes in patients with advanced HER-2 positive gastric cancer treated with taxanes-based chemotherapy and trastuzumab.

Study	Phase	Treatment	n	PFS (mo)	OS (mo)	ORR (%)	Secondary resectability (%)	Reference
Mitsuy et al.	II	T + DC + S1	16	NR	NR	93.8	56	(19)
Mondaca et al.	II	T + mDCF	26	13	24.9	65	–	(21)
Roviello et al.	II	T+ DOF	15	9.2	19.4	60	–	(20)
Current study	Retrospective	TPFT	21	9.3	19	68.4	29.4	–

C, cisplatin; D, docetaxel; mDCF, modified DCF regimen; F, 5-FU; PFS, progression-free survival; mo, months; NR, not reached; O, oxaliplatin; ORR, objective response rate; OS, overall survival; T, trastuzumab. TPFT, Taxane Platine 5-FU trastuzumab (composed of 75% of mDCF treatment).

In our TanDHER study, an objective response was observed in 68.2% in the T group, similar to 60 to 94% of phase II trials (19–21). Besides, the secondary resection of the primary tumor was performed in five among 17 patients (29%) in the T group, compared to one patient in the S group in the population of analysis. Mitsui et al. have also shown a high secondary resection rate with 9 of 16 patients (56%) undergoing surgery of their metastasis and primary tumor.

Over the last decade, published data are in favor of a conversion therapy after chemotherapy in selected patients and seems to offer longer survival than chemotherapy alone (26). For example, Yamaguchi et al. have shown that conversion therapy was performed in 77 patients in a retrospective study including in 259 advanced GEA patients with systemic chemotherapy. The median survival time was 30.5 months in patients who underwent surgical resection, as opposed to 11.3 months in those who received chemotherapy alone (27). In our study, we observed a prolonged PFS and OS in the case of surgical resection of the primary tumor. This therapeutic strategy seems to be an interesting option in advanced GEA and should be discussed in multidisciplinary board in good responders with oligometastatic disease.

Our study has several limits naturally related to its size and retrospectively analyzed results. However, our results are highly comparative with previous phase II and retrospective data and support the potential interest of the addition of taxane at first-line in metastatic GEA patients.

Besides, anti-PD-1 immunotherapy is a slowly gaining field in advanced GEA in first-line treatment in combination with chemotherapy. The recent results of the CheckMate 649 and ATTRACTION-4 clinical trials demonstrated efficacy in advanced HER2-negative GEA (28, 29). In HER2-positive GEA, phase II and III trials are still ongoing, evaluating the addition of anti-PD-1 therapy (as monotherapy or in combination with anti-CTLA-4) to trastuzumab +/- platin-based chemotherapy (NCT02901301, NCT03615326, NCT03409848) (30, 31). Recently, the prespecified intermediate analysis of Keynote 811 phase III study, demonstrated an improvement in ORR with the addition of pembrolizumab to the PFT regimen (74.4% vs 51.9%; p=0.00006) (30). Furthermore, the combination of immune checkpoints inhibitors to taxanes-based chemotherapy enhanced the anti-tumor activity in preclinical data. Recent findings describe their ability to stimulate innate immune response and to promote adaptive antitumor immune response, which can explain the additive effect of the two classes of drugs (32). Thus, the association of anti-PD-(L)1 monoclonal antibody with taxanes-based chemotherapy was approved for the first-line treatment of unresectable triple-negative breast cancer or metastatic squamous NSCLC (33–35). In gastrointestinal oncology, a phase II study of DCFm regimen in combination with atezolizumab in unresectable locally advanced squamous cell anal carcinoma is still ongoing (36). At the interim analysis, no safety issue is described (publication under review). Based on these observations, adding anti-PD-(L)1 immunotherapy to trastuzumab plus taxanes-based chemotherapy could be considered for further investigations.

In conclusion, our data suggest that adding docetaxel to standard treatment in first-line advanced HER2 positive gastroesophageal adenocarcinoma is active in selective patients, with significantly longer PFS and OS compared to the standard regimen. Conversion therapy may be considered in oligometastatic patients with good responses.

## Data Availability Statement

The original contributions presented in the study are included in the article/**Supplementary Material**. Further inquiries can be directed to the corresponding author.

## Ethics Statement

The studies involving human participants were reviewed and approved by Clinical Ethics Committee, CHRU Besancon. The patients/participants provided their written informed consent to participate in this study.

## Author Contributions

EO, JH, SK, and DV contributed to conception and design of the study. EO and SK organized the database. JH performed the statistical analysis. EO wrote the first draft of the manuscript. SK, JH, and DV wrote sections of the manuscript. All authors contributed to manuscript revision, read, and approved the submitted version.

## Conflict of Interest

The authors declare that the research was conducted in the absence of any commercial or financial relationships that could be construed as a potential conflict of interest.

## Publisher’s Note

All claims expressed in this article are solely those of the authors and do not necessarily represent those of their affiliated organizations, or those of the publisher, the editors and the reviewers. Any product that may be evaluated in this article, or claim that may be made by its manufacturer, is not guaranteed or endorsed by the publisher.
