# Supplementary material for: Interest of the Addition of Taxanes to Standard Treatment in First-Line Advanced HER2 Positive Gastroesophageal Adenocarcinoma in Selective Patients
Source: Front Oncol. 2022 Mar 7;12:763926. doi: 10.3389/fonc.2022.763926 (PMC8948436; doi:10.3389/fonc.2022.763926)
Supplement: Supplementary file 1 [file Table_1.docx]

Supplementary Material

# Supplementary Figures and Tables

**Supplementary Table 1**: Baseline characteristics of patients in the overall population.

*S group: Standard regimen. T group: TPFT regimen. Abbreviations: FISH: Fluorescence in situ Hybridization. HER2: Human Epidermal Growth Factor Receptor 2. PS: Performance status.*

|  |  | | **Total  (n=65)** | | | | **S group (n=41)** | | | | **T group (n=24)** | | |  | |  |
| --- | --- | --- | --- | --- | --- | --- | --- | --- | --- | --- | --- | --- | --- | --- | --- | --- |
|  |  | | **n** | | **%** | | **n** | | **%** | | **n** | | **%** | **p-value** | |  |
| **Age** | Median | | 68.65 | |  | | 72.8 | |  | | 64.49 | |  | **0.0152** | |  |
|  | Range | | 30.34-90.56 | | | | 30.34-90.56 | | | | 39.00-79.30 | | |  | |  |
| **Gender** | Male | | 56 | | 86.15 | | 35 | | 85.37 | | 21 | | 87.5 | 1 | |  |
|  | Female | | 9 | | 13.85 | | 6 | | 14.63 | | 3 | | 12.5 |  | |  |
| **ECOG PS** | 0-1 | | 55 | | 85.94 | | 32 | | 80 | | 23 | | 95.83 | 0.1361 | |  |
|  | 2 | | 9 | | 14.06 | | 8 | | 20 | | 1 | | 4.17 |  | |  |
|  | Missing | | 1 | |  | | 1 | |  | | 0 | |  |  | |  |
| **Initial diagnosis** |  | |  | |  | |  | |  | |  | |  |  | |  |
| **Primary tumor** | Stomach | | 41 | | 63.08 | | 28 | | 68.29 | | 13 | | 54.17 | 0.2548 | |  |
|  | Gastro-Esophageal junction/ Lower esophagus | | 24 | | 36.92 | | 13 | | 31.71 | | 11 | | 45.83 |  | |  |
| **Metastasis status** | Synchronous | | 49 | | 75.38 | | 27 | | 65.85 | | 22 | | 91.67 | **0.0197** | |  |
|  | Metachronous | | 16 | | 24.62 | | 14 | | 34.15 | | 2 | | 8.33 |  | |  |
| **Metastasis location** |  | |  | |  | |  | |  | |  | |  |  | |  |
| **Peritoneal carcinomatosis** | No | | 54 | | 83.08 | | 36 | | 87.8 | | 18 | | 75 | 0.3035 | |  |
|  | Yes | | 11 | | 16.92 | | 5 | | 12.2 | | 6 | | 25 |  | |  |
| **Bones** | No | | 59 | | 90.77 | | 38 | | 92.68 | | 21 | | 87.5 | 0.6616 | |  |
|  | Yes | | 6 | | 9.23 | | 3 | | 7.32 | | 3 | | 12.5 |  | |  |
| **Lung** | No | | 52 | | 80 | | 32 | | 78.05 | | 20 | | 83.33 | 0.7527 | |  |
|  | Yes | | 13 | | 20 | | 9 | | 21.95 | | 4 | | 16.67 |  | |  |
| **Brain** | No | | 62 | | 95.38 | | 38 | | 92.68 | | 24 | | 100 | 0.2904 | |  |
|  | Yes | | 3 | | 4.62 | | 3 | | 7.32 | | 0 | | 0 |  | |  |
| **Liver** | No | | 25 | | 38.46 | | 16 | | 39.02 | | 9 | | 37.5 | 0.903 | |  |
|  | Yes | | 40 | | 61.54 | | 25 | | 60.98 | | 15 | | 62.5 |  | |  |
| **Other metastasis** | No | | 61 | | 93.85 | | 41 | | 100 | | 20 | | 83.33 | 0.0157 | |  |
|  | Yes | | 4 | | 6.15 | | 0 | | 0 | | 4 | | 16.67 |  | |  |
| **Pathology Characteristics** | | |  | |  | |  | |  | |  | |  |  | |  |
| **Histology** | Well | | 14 | | 25 | | 10 | | 28.57 | | 4 | | 19.05 | 0.4256 | |  |
|  | Moderately/Undifferentiated | | 42 | | 75 | | 25 | | 71.43 | | 17 | | 80.95 |  | |  |
|  | Missing | | 9 | |  | | 6 | |  | | 3 | |  |  | |  |
| **HER2 Status** | HER2 3+ | | 58 | | 89.23 | | 36 | | 87.8 | | 22 | | 91.67 | 1 | |  |
|  | HER2 2+/FISH+ | | 7 | | 10.77 | | 5 | | 12.2 | | 2 | | 8.33 |  | |  |
| **Surgical management of primary tumor** | | |  |  | |  | |  | |  | |  | |  |  |  |
|  | No |  | 48 | 73.85 | | 26 | | 63.41 | | 22 | | 91.67 | | **0.0124** |  |  |
|  | Yes |  | 17 | 26.15 | | 15 | | 36.59 | | 2 | | 8.33 | |  |  |  |
|  |  |  |  |  | |  | |  | |  | |  | |  |  |  |
|  |  |  |  |  | |  | |  | |  | |  | |  |  |  |

**Supplementary Table 2**. Univariate and multivariate Cox regression for OS in the population of analysis.

*Population of analysis: patients with ECOG PS 0 - 1 and synchronous metastasis. Abbreviations: HR: Hazard Ratio. PS: Performance Status.*

|  |  | **Univariate Cox regression** | | | | **Multivariate Cox regression** | | | |
| --- | --- | --- | --- | --- | --- | --- | --- | --- | --- |
|  |  | **n (events)** | **HR** | **95%CI** | **p-value** | **n (events)** | **HR** | **95%CI** | **p-value** |
| **Gender** | **Male** | 34 (25) | 1 |  | 0.9211 |  |  |  |  |
|  | **Female** | 6 (5) | 1.05 | 0.40-2.78 |  |  |  |  |  |
| **Age at treatment initiation** | **Continuous** | 40 (30) | 1.02 | 0.99-1.05 | 0.2287 |  |  |  |  |
|  | **<70** | 22 (15) | 1 |  | **0.0717** | 22 (15) | 1 |  | 0.4884 |
|  | ≥**70** | 18 (15) | 1.96 | 0.94-4.08 |  | 18 (15) | 1.33 | 0.59-2.99 |  |
| **ECOG PS** | **0** | 21 (15) | 1 |  | **0.0926** | 21 (15) | 1 |  | 0.1175 |
|  | **1** | 19 (15) | 1.9 | 0.90-4.00 |  | 19 (15) | 1.87 | 0.86-4.09 |  |
| **Initial Diagnosis** |  |  |  |  |  |  |  |  |  |
| **Primary tumor** | **Stomach** | 27 (24) | 1 |  | 0.1998 |  |  |  |  |
|  | **GEJ/lower esophagus** | 13 (6) | 0.55 | 0.22-1.37 |  |  |  |  |  |
| **Histology** | **Well** | 10 (7) | 1 |  | 0.5069 |  |  |  |  |
|  | **Moderately/**  **undifferentiated** | 25 (22) | 1.34 | 0.57-3.14 |  |  |  |  |  |
| **Metastasis location** |  |  |  |  |  |  |  |  |  |
| **Peritoneum** | **No** | 31 (25) | 1 |  | 0.6824 |  |  |  |  |
|  | **Yes** | 9 (5) | 1.23 | 0.46-3.24 |  |  |  |  |  |
| **Lymph nodes** | **No** | 17 (12) | 1 |  | 0.4992 |  |  |  |  |
|  | **Yes** | 23 (18) | 1.29 | 0.62-2.68 |  |  |  |  |  |
| **Lung** | **No** | 32 (23) | 1 |  | 0.9296 |  |  |  |  |
|  | **Yes** | 8 (7) | 0.96 | 0.41-2.26 |  |  |  |  |  |
| **Liver** | **No** | 13 (9) | 1 |  | 0.6309 |  |  |  |  |
|  | **Yes** | 27 (21) | 1.22 | 0.55-2.69 |  |  |  |  |  |
| **Taxanes** | **No** | 19 (17) | 1 |  | **0.0378** | 19 (17) | 1 |  | 0.0702 |
|  | **Yes** | 21 (13) | 0.45 | 0.21-0.96 |  | 21 (13) | 0.47 | 0.21-1.06 |  |

**Supplementary Table 3**. Univariate and multivariate logistic regression to estimate the probability to have taxanes-based chemotherapy in the population of analysis.

*Population of analysis: patients with ECOG PS 0 - 1 and synchronous metastasis. Abbreviations: CI: Confidence Interval. OR: Odds Ratio. PS: Performance Status.*

|  |  | **Univariate analysis** | | | | **Multivariate analysis** | | |
| --- | --- | --- | --- | --- | --- | --- | --- | --- |
|  |  | **n**  **(pts with taxanes)** | **OR** | **95%CI** | **p-value** | **OR** | **95%CI** | **p-value** |
| **Gender** | **Male** | 34 (18) | 1 |  | 0.8941 |  |  |  |
|  | **Female** | 6 (3) | 0.89 | 0.16-5.04 |  |  |  |  |
| **Age at treatment initiation** | **Continuous** | 40 (21) | 95 | 0.90-1.01 | 0.0893 | 0.95 | 0.90-1.01 | 0.1063 |
|  |  |  |  |  |  |  |  |  |
|  | **<70** | 22 (14) | 1 |  | 0.123 |  |  |  |
|  | **≥70** | 18 (7) | 0.36 | 0.10-1.32 |  |  |  |  |
| **ECOG PS** | **0** | 21 (12) | 1 |  | 0.5371 |  |  |  |
|  | **1** | 19 (9) | 0.68 | 0.19-2.35 |  |  |  |  |
| **Initial diagnosis** |  |  |  |  |  |  |  |  |
| **Primary tumor** | **Stomach** | 27 (12) | 1 |  | 0.1482 | 1 |  | 0.1856 |
|  | **Gastro-Esophageal junction/ Lower esophagus** | 13 (9) | 2.81 | 0.69-11.42 |  | 2.65 | 0.63-11.21 |  |
| **Metastasis location** |  |  |  |  |  |  |  |  |
| **Peritoneal carcinomatoses** | **No** | 31 (15) | 1 |  | 0.3395 |  |  |  |
|  | **Yes** | 9 (6) | 2.13 | 0.45-10.10 |  |  |  |  |
| **Metastatic nodes** | **No** | 17 (11) | 1 |  | 0.1877 |  |  |  |
|  | **Yes** | *23 (10)* | 0.42 | 0.12-1.53 |  |  |  |  |
| **Lung** | **No** | 32 (18) | 1 |  | 0.3483 |  |  |  |
|  | **Yes** | *8 (3)* | 0.47 | 0.10-2.30 |  |  |  |  |
| **Liver** | **No** | 13 (7) | 1 |  | 0.9059 |  |  |  |
|  | **Yes** | 27 (14) | 0.93 | 0.25-3.48 |  |  |  |  |

**Supplementary Table 4**: Response rate according to RECIST criteria in the population of analysis.

*Population of analysis: patients with ECOG PS 0 - 1 and synchronous metastasis. S group: Standard regimen. T group: TPFT regimen.*

|  | **S group (n=19)** | **T group (n=21)** | **p-value** |
| --- | --- | --- | --- |
| **Best response** | **n (%)** | **n (%)** |  |
| **Objective Response Rate** | 9 (47.37) | 13 (68.42) | 0.1888 |
| Complete response | 0 (0) | 0 (0) |  |
| Partial response | 9 (47.37) | 13 (68.42) |  |
| **Disease Control Response** | 12 (63.16) | 17 (89.47) | 0.1245 |
| Stable disease | 3 (15.79 | 4 (21.05) |  |
| **Progression Disease** | 7 (36.84) | 2 (10.43) |  |
| **Missing** | 0 | 2 |  |

**Supplementary Table 5**: Conversion therapy after chemotherapy in patients with disease control rate in the population of analysis.

*Conversion therapy is defined as surgery or other local treatment of primary tumor or metastasis. Population of analysis: patients with ECOG PS 0 - 1 and synchronous metastasis. S group: Standard regimen. T group: TPFT regimen.*

|  |  | **S group (n=12)** | | **T group (n=17)** | |  |
| --- | --- | --- | --- | --- | --- | --- |
|  |  | **n** | **%** | **n** | **%** | **p-value** |
| **Metastasis surgery** | **No** | 11 | 91.67 | 14 | 82.35 | 0.6221 |
|  | **Yes** | 1 | 8.33 | 3 | 17.65 |  |
| **Resection of primary tumor** | **No** | 11 | 91.67 | 12 | 70.59 | 0.3544 |
|  | **Yes** | 1 | 8.33 | 5 | 29.41 |  |
| **Other local treatment** | **No** | 12 | 100 | 16 | 94.12 | 1 |
|  | **Yes** | 0 | 0 | 1 | 5.88 |  |

**Supplementary Table 6**: Characteristics of patients who underwent conversion surgery

*Cycle corresponding to the number of cycles of chemotherapy before surgery, the pathological response was evaluated with Becker classification (31). Abbreviations: DCF: standard regimen with docetaxel, cisplatin, and 5-FU + trastuzumab, LN: lymph nodes, LND: lymph nodes dissection, LS: Lewis Santy surgery, MD: missing data, mo: months, ORR: objective response rate, OS: overall survival, PC: peritoneal carcinomatosis, PFS: progression-free survival, PH partial hepatectomy, PR: partial response, T: trastuzumab, RFA: radiofrequency ablation, SBTR: stereotactic body radiotherapy, TG: total gastrectomy; D2: D2 lymph node dissection.*

| **Case** | **Age at diagno-sis** | **Non -curative factors** | **Location** | **HER2** | **Treat-ment** | **ORR** | **Cycle** | **Surgery (+/- other local treatment)** | **Pathologic**  **response** | | **Adjuvant treatment** | **Recur-rence** | **PFS (mo)** | **OS (mo)** | **Time without outcome (mo)** | |
| --- | --- | --- | --- | --- | --- | --- | --- | --- | --- | --- | --- | --- | --- | --- | --- | --- |
|  |  |  |  |  |  |  |  |  | **Tumor** | **Metas-tasis** |  |  |  |  |  |  |
| 1 | 39 | LN, liver | Stomach | 3+ | T + DCF | PR | 4 | TG/LND/PH | G3 | G3 | Yes: T-DCF | Yes | 8.2 | 17.7 | - |  |
| 2 | 54 | LN, lung | Lower oeso-phagus | 3+ | T + mDCF | PR | 8 | LS/LND/pulmonary wedge resection | MD | MD | Yes: T-capecitabine | No | - | - | 35.4 |  |
| 3 | 54 | Liver | Lower oeso-phagus | 3+ | T + mDCF | PR | 8 | LS/LND/RFA (liver) | MD | - | No | Yes | 17.5 | - | 27.6 |  |
| 4 | 57 | Liver | Lower oeso-phagus | 3+ | T + DCF | PR | 4 | LS/LND/PH | G2 | G2 | Yes: T | No | - | - | 90.5 |  |
| 5 | 76 | PC | Stomach | 3+ | T + mDCF | PR | 8 | TG/LND | MD | CR peritoneum | Yes: T-DCF 🡪 T | No | - | 27 | - |  |
| 6 | 80 | LN | Lower oeso-phagus | 2+ | T + FOLFOX | PR | 16 | LS/LND/SBTR (LN) | G3 | - | No | Yes | 25.7 | 41.5 | - |  |

**Supplementary Table 7**: Evaluation of toxicity in the population of analysis.

*Dose reduction corresponds to the rate of patients with a dose reduction of one of the chemotherapy molecules during the entire course of treatment. Early dose reduction corresponds to the rate of patients with a dose reduction of one of the chemotherapy molecules during the first 3 cycles of treatment.*

|  | **Total**  **(n=40)** | | **S group**  **(n=19)** | | **T group**  **(n=21)** | |  |
| --- | --- | --- | --- | --- | --- | --- | --- |
|  | **n** | **%** | **n** | **%** | **n** | **%** | **p-value** |
| Median number of cycles (range) | 8 (1-16) | | 9 (1-15) | | 8 (2-16) | | 0.9566 |
| Dose reduction | 27 | 68 | 14 | 74 | 13 | 62 | 0.427 |
| Early dose reduction (cycle 1 to 3) | 13 | 33 | 7 | 37 | 6 | 29 | 0.577 |
| Toxic death | 0 | | 0 | | 0 | |  |
